# Sialidases From *Clostridium perfringens* and Their Inhibitors

**DOI:** 10.3389/fcimb.2019.00462

**Published:** 2020-01-10

**Authors:** Yan-hua Wang

**Affiliations:** State Key Laboratory of Veterinary Etiological Biology, Lanzhou Veterinary Research Institute, Chinese Academy of Agricultural Sciences, Lanzhou, China

**Keywords:** *C. perfringens*, sialidase, molecular properties, regulatory mechanism, pathogenesis, sialidase inhibitor

## Abstract

*Clostridium perfringens* is an important human and animal pathogen that is the primary causative agent of necrotizing enteritis and enterotoxemia in many types of animals; it causes traumatic gas gangrene in humans and animals and is associated with cases of food poisoning in humans. *C. perfringens* produces a variety of toxins as well as many enzymes, including three sialidases, NanH, NanI, and NanJ. Sialidases could be important virulence factors that promote the pathogenesis of *C. perfringens*. Among them, NanI promotes the colonization of *C. perfringens* in the intestinal tract and enhances the cytotoxic activity and association of several major *C. perfringens* toxins with host cells. In recent years, studies on the structure and functions of sialidases have yielded interesting results, and the functions of sialic acid and sialidases in bacterial pathogenesis have become a hot research topic. An in-depth understanding and additional studies of sialidases will further elucidate mechanisms of *C. perfringens* pathogenesis and could promote the development and clinical applications of sialidase inhibitors. This article reviews the structural characteristics, expression regulation, roles of sialidases in *C. perfringens* pathogenesis, and effects of their inhibitors.

## Introduction

*Clostridium perfringens* is a gram-positive anaerobic bacillus that is widely present in nature, especially in the soil and intestines of human and animals. *C. perfringens* can infect humans and various animals (primarily cows and sheep), causing food poisoning in humans, necrotizing enteritis, and enterotoxemia in animals and traumatic gas gangrene in both humans and animals, as well as other diseases. These diseases not only seriously threaten the health of humans and animals but also cause enormous economic losses (Parent et al., 2017; Silva et al., 2018).

At present, more than 20 *C. perfringens* toxins have been identified, and there may be additional toxins that have yet to be identified (Hatheway, 1990; Petit et al., 1999; Amimoto et al., 2007; Keyburn et al., 2008; Yonogi et al., 2014; Irikura et al., 2015; Mehdizadeh Gohari et al., 2016). Based on the primary toxins produced by *C. perfringens* and differences in pathogenesis among strains, *C. perfringens* strains are divided into seven types according to a recent revision (Type A–G, Table 1; Rood et al., 2018). The α-toxin gene is the most prevalent toxin gene carried among *C. perfringens* and is encoded on chromosome. The genes encoding the beta, epsilon, iota, and NetB toxins are plasmid-borne, whereas CPE can be encoded either on the chromosome or on a plasmid (Hassan et al., 2015). Of the seven types of *C. perfringens*, the type F strains are considered the most common pathogen in humans (Rood et al., 2018).

**Table 1:** The *C. perfringens* toxin-based typing scheme.

**Toxinotype** **(toxin gene)**	**α-toxin** **(*plc* or *cpa*)**	**β-toxin** **(*cpb*)**	**ε-toxin** **(*etx*)**	**ι-toxin** **(*iap* and *ibp*)**	**CPE** **(*cpe*)**	**NetB** **(*netB*)**
A	+	–	–	–	–	–
B	+	+	+	–	–	–
C	+	+	–	–	±	–
D	+	–	+	–	±	–
E	+	–	–	+	±	–
F	+	–	–	–	+	–
G	+	–	–	–	–	+

*+ Means the toxin can be produced by the toxinotype; – means the toxin cannot be produced by the toxinotype; and ± means the toxin may or may not be produced by the toxinotype*.

So far many studies have shown that sialidases play important roles in the pathogenesis of pathogenic bacteria, providing nutrition for bacteria and promoting bacterial colonization, bacterial adhesion, bacterial internalization, biofilm formation, and the binding of toxins to host cells (Traving and Schauer, 1998; Vimr et al., 2004; Tong et al., 2005; Pastoriza Gallego and Hulen, 2006; Soong et al., 2006; Severi et al., 2007; Parker et al., 2009; Thompson et al., 2009; Trappetti et al., 2009; Banerjee et al., 2010; King, 2010; Honma et al., 2011; Li et al., 2011; Brittan et al., 2012; Lewis and Lewis, 2012; Awad et al., 2016; Blanchette et al., 2016). Recently, a study found that NanA, NanB, and NanC increased the interaction of *S. pneumoniae* with human airway epithelial cells (Janesch et al., 2018). Some pathogens also use sialic acid to coat their cell surface, flagella, capsule polysaccharides, or lipopolysaccharides, concealing themselves to evade the host immune system (Severi et al., 2007). Free sialic acid also participates in capsule formation in *Neisseria meningitidis, Escherichia coli*, and *Porphyromonas gingivalis* and defends cells against the immune responses of the host (Vimr et al., 2004; Allen et al., 2005), although the mechanism associated with this activity is unclear. Meningococcal capsule can block the killing effect of human serum, which may be due to sialic acids concealing the membrane attack complex on the bacterial cell membrane (Vimr and Lichtensteiger, 2002). In addition, a *Vibrio cholerae* sialidase can hydrolyze ganglioside on the surfaces of intestinal mucosal epithelial cells, and ganglioside GM1 binds the enterotoxin of *V. cholerae* to disrupt the normal function of cellular ion channels, leading to dehydration and other symptoms in the human body (Vimr and Lichtensteiger, 2002). In a recent study, sialidases from microorganisms in the cervix and vagina were observed to modify gonococci and enhance the successful transmission of the pathogen to men (Ketterer et al., 2016). Therefore, sialidases play significant roles in the survival and pathogenesis of bacteria. Furthermore, recent studies have shown that a sialidase deficiency in *P. gingivalis* can weaken the activation of CR3 in macrophages, reduce the inhibition of lncRNA GAS5 by CR3, and induce less miR-21 and more IL-12 production in macrophages. These results suggest that the inhibition of sialidase activity in *P. gingivalis* renders the bacteria easier to be cleared by macrophages (Yang et al., 2018), and this discovery will open up a new direction for the prevention and treatment of chronic periodontitis. Sialidases are also a marker of some diseases (Liu et al., 2018). Similarly, sialidases can also contribute to important steps in the pathogenesis of *C. perfringens*, and studies on this activity have made substantial progress. This article reviews the structural characteristics, expression regulation, and roles of sialidases in the pathogenesis of *C. perfringens*.

## Sialidases

Sialidases, also known as neuraminidases (E.C.3.2.1.18), hydrolyze the α-glycoside bond of the terminal sialic acid in glycoproteins and glycolipids to produce free sialic acid (Traving and Schauer, 1998; Vimr et al., 2004; Severi et al., 2007; Lewis and Lewis, 2012). Hydrolytic-sialidases usually have wide substrate specificity and cleave α2-3-, α2-6-, and α2-8-linked terminal sialic acids (Juge et al., 2016). Sialidases are the key enzymes responsible for the catabolism of oligosaccharides containing sialic acid (Lewis and Lewis, 2012).

Sialidases participate in cell metabolism, adhesion, proliferation, immune functions, and infectious processes under various pathological and physiological conditions (Imai and Kawaoka, 2012; Varki and Gagneux, 2012; Walther et al., 2013; de Graaf and Fouchier, 2014; Arabyan et al., 2017; Miyagi et al., 2018). Sialidases are known to be produced by a variety of viruses and bacterial pathogens, including influenza viruses, *V. cholerae, S. pneumoniae, E. coli, Staphylococcus aureus*, and *C. perfringens* (Traving and Schauer, 1998; Vimr et al., 2004; Severi et al., 2007; Nishiyama et al., 2018). Therefore, sialidases are virulence factors involved in the pathogenesis of infections (Rohmer et al., 2011; Lewis and Lewis, 2012; Li et al., 2016).

## Molecular and Structural Characteristics of Sialidases From *C. perfringens*

*C. perfringens* produces three sialidases, NanH, NanI, and NanJ (Li and McClane, 2014a), all of which are encoded by genes located on different regions of the chromosome (Shimizu et al., 2002; Myers et al., 2006). They are classified in the GH family 33 (GH33) of the CAZy classification (Lombard et al., 2014). Sequencing analysis of *C. perfringens* sialidase ORFs showed that the similarity of *nanJ, nanI*, and *nanH* gene sequences from different *C. perfringens* strains ranges from 96 to 100, 98 to 100, and 93 to 100%, respectively (Shimizu et al., 2002; Myers et al., 2006; Li et al., 2011). The *nanI* and *nanJ* genes have 2–3 hypothetical transcription initiation sites (Therit et al., 2015), which are located within 500 bp of the start codon of these genes. These diverse promoters allow for sialidase expression to be regulated by a variety of different regulatory factors (Therit et al., 2015). The *nanI* and *nanJ* promoter regions and the hypothetical promoter region of *nanH* in strain SM101 contain a conserved 14-bp sequence (a consensus sequence of 5′-GAAAAATATTTTC-3′). These conserved repetitive sequences are located in the transcription initiation region of the promoters and may function as recognition sequences of transcription factors that regulate the production of *C. perfringens* sialidases.

**Figure 1:**
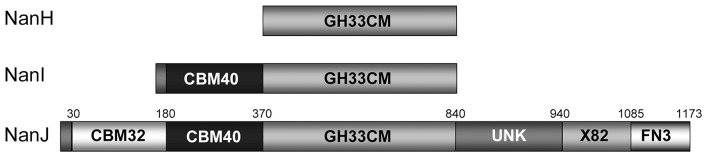
Modular organization of the clostridial sialidases (Boraston et al., 2007) Copyright © 2007, with permission from American Chemical Society. Amino acid numbers corresponding to the module boundaries are shown above the schematic of NanJ (Boraston et al., 2007).

**Figure 2:**
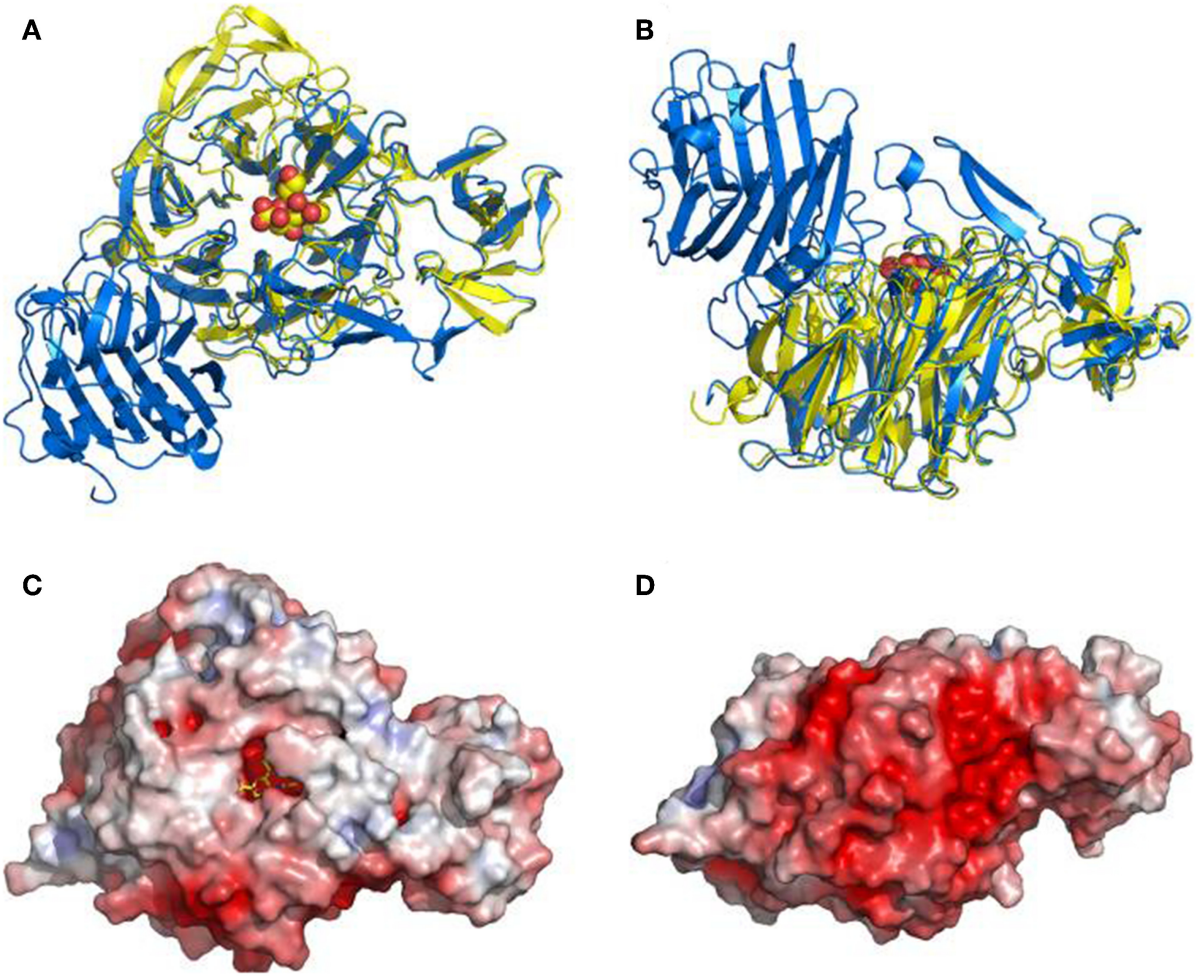
Overall structure of the NanI sialidase. Reprinted from Newstead et al. (2008) Copyright © 2008, with permission from the American Society for Biochemistry and Molecular Biology, Inc. **(A,B)** Represent orthogonal views of a comparison of the fold of NanI (yellow) with the leech sialidase (blue). Sialic acid is drawn as spheres to locate the active site. **(C,D)** Show a surface representation of NanI, in the same orientations as above, colored according to electrostatic potential from −7 to +7 *k*T/e, calculated using APBS.

The small protein NanH (43 kDa) lacks a signal peptide and is located in the cytoplasm during logarithmic growth (Li et al., 2011; Li and McClane, 2014a). In contrast, the NanI (77 kDa) and NanJ (129 kDa) are secreted into the extracellular matrix (Li and McClane, 2014a). These three sialidases have similarities and differences in structure (Figure 1). The amino acid sequences of the catalytic domains of these sialidases are conserved among sialidases (Boraston et al., 2007). NanH contains only the catalytic domain, while NanI has a carbohydrate-binding domain (CBM40) in addition to the catalytic domain (Boraston et al., 2007). The structure of NanJ is complex, consisting of a catalytic domain, two carbohydrate binding domains (CBM32 and CBM40), and three additional auxiliary regions (Boraston et al., 2007). These carbohydrate-binding regions increase the binding affinity of NanI and NanJ toward their polyvalent substrates. The characteristics of sialidases from *C. perfringens* are summarized in Table 2. The structure of the catalytic domain (residues 243–694) of NanI has been studied (Newstead et al., 2004, 2008), showing that NanI folds into two distinct domains: a regular six-blade β-propeller catalytic domain formed by residues 243–359 and 429–691 and a small β-barrel domain formed by residues 360–428 (Figure 2) (Newstead et al., 2008). The carbohydrate binding domains CBM32 and CBM40 of NanJ recognize Gal and Neu5Ac, respectively (Boraston et al., 2007).

**Table 2:** Characterized *C. perfringens* sialidases.

**Protein name**	**Bacterial strain**	**Domains**	**Substrates tested**	**References**
NanH	*C. perfringens* ATCC 10543	GH33	4MU-Neu5Ac	Chien et al., 1996
NanI	*C. perfringens* strain 13	CBM40, GH33	Fetuin, bovine submaxillary mucin, colominic acid, bovine brain gangliosides can also hydrate 2-deoxy- 2,3-dehydro-Neu5Ac acid to Neu5Ac	Shimizu et al., 2002; Minami et al., 2013
NanJ	*C. perfringens* strain 13/ATCC 13124II	CBM32, CBM40, GH33	Only the CBMs are characterized	Boraston et al., 2007

## The Enzymatic Characteristics of Three *C. perfringens* Sialidases

Most *C. perfringens* strains produce three sialidases, NanH, NanI, and NanJ, while some produce only one or two of these sialidases. The activity of NanI typically accounts for 70% of the total extracellular sialidase activity in strains that produce the three types of sialidase (Chiarezza et al., 2009; Li and McClane, 2014a). To study the properties of the three enzymes, those sialidase genes were inactivated in *C. perfringens* type D strain CN3718 to construct a series of isogenic mutants (Li and McClane, 2014a). The results showed that NanJ and NanH were most active at 37 and 43°C, respectively. These two sialidases showed low activity at 48°C, while the activity of NanI increased steadily as the temperature increased to 48°C. At 25°C, the activity of the three sialidases was relatively low. After incubating at 60°C for 5 min in the same buffer, the loss of enzyme activity of NanI in the culture supernatant was ~50%, while that of NanJ and NanH exceeded 80%. Thus, compared to NanJ and NanH, NanI exhibits higher heat resistance (Li and McClane, 2014a).

The activity of the three *C. perfringens* sialidases was the highest at pH 5, and each enzyme exhibits different sensitivity to various metal ions (Li and McClane, 2014a). Fe^2+^, Mn^2+^, and Mg^2+^ were found to enhance NanI enzyme activity, while Fe^3+^ and Zn^2+^ decreased NanI enzyme activity. More metal ions (Fe^2+^, Mn^2+^, Co^2+^, Mg^2+^, Ni^2+^, and Zn^2+^) increased NanJ activity, only Fe^3+^ decreased NanJ activity. In contrast, for NanH activity, all metal ions mentioned above, except Mg^2+^, were found to decrease its activity. Furthermore, unlike sialidases from *Streptococcus*, those of *C. perfringens* are sensitive to p-chloromethylbenzoate, which reacts with the sulfhydryl groups of proteins. Among the three enzymes, NanJ is the least sensitive to p-chloromethylbenzoate, and 10 mM p-chloromethylbenzoate can inhibit the activity of all three sialidases. EDTA has a minor inhibitory effect on NanI, while NanH and NanJ are not affected (Li and McClane, 2014a). These results further confirm differences between the activities of the *C. perfringens* sialidases.

The three *C. perfringens* sialidases exhibit different substrate preferences. NanI showed preferential activity in the order of α-2,3 > α-2,6 > α-2,8 linkages. NanJ activities showed a preference for α-2,6 > α-2,8 > α-2,3 sialic acid linkages. NanH activities showed a preference for α-2,8 > α-2,3 > α-2,6 linkages (Li and McClane, 2014a). The diversity of these preferences suggests that these three sialidases in combination can hydrolyze and release free sialic acid from complex substrates (Li and McClane, 2014a).

## Regulation of *C. perfringens* Sialidase Expression

**Figure 3:**
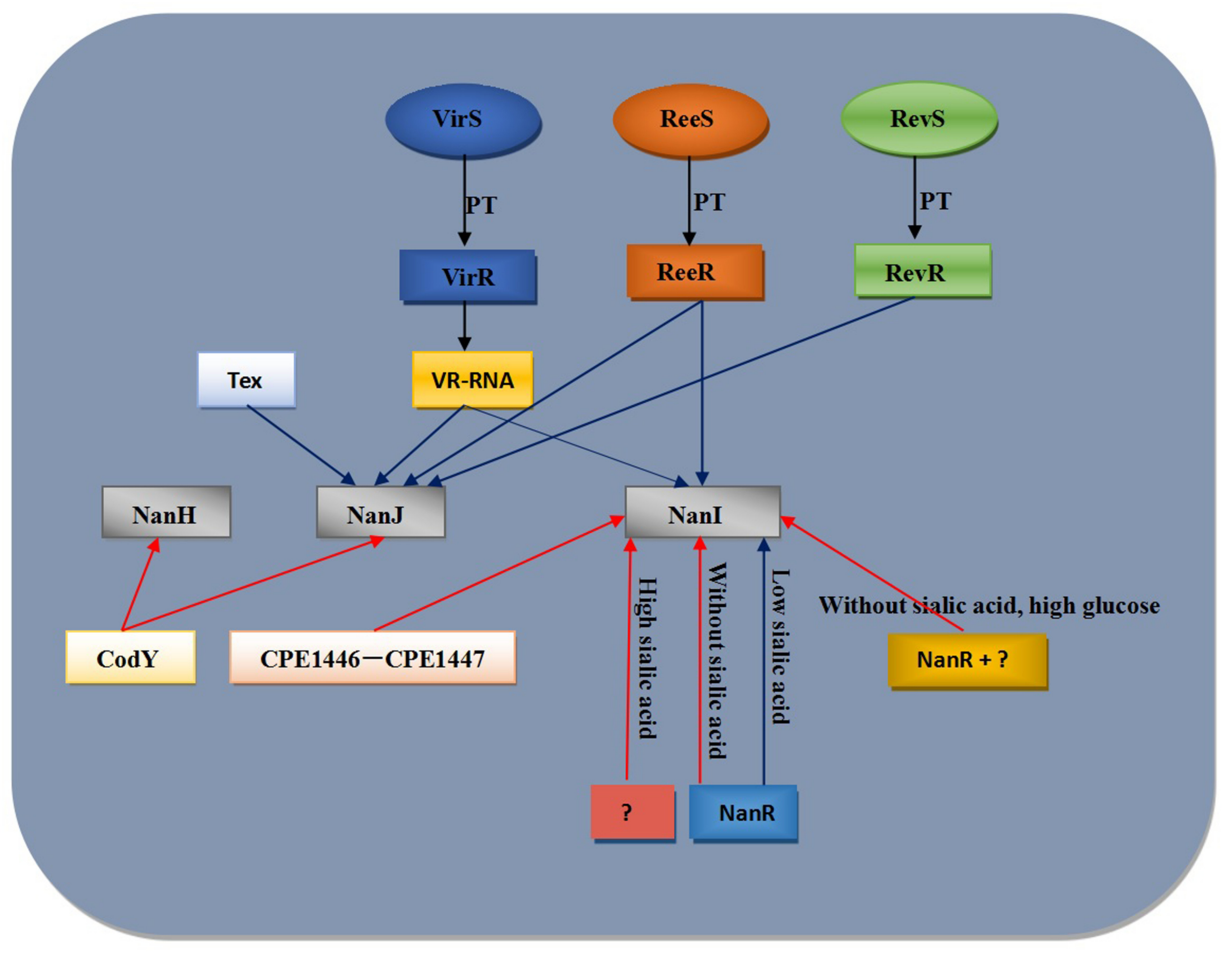
Model for regulation of *C. perfringens* sialidase expression. Blue lines indicate positive regulation of sialidase expression while red lines indicate negative regulation regulation of sialidase expression. PT, phosphotransfer.

Previous studies have reported that the addition of free sialic acid to culture medium could induce extracellular sialidase activity in *C. perfringens* (Nees and Schauer, 1974), suggesting the presence of a specific regulatory system that responds to sialic acid and regulates the transcription of sialidase-encoding genes. Subsequent studies showed that multiple proteins are involved in regulating sialic acid-related gene expression (Figure 3). It has been demonstrated that VirS/VirR, ReeS/ReeR, RevS/RevR, CodY, and NanR directly or indirectly affect the production of sialidases (Ohtani et al., 2010; Hiscox et al., 2011, 2013; Li et al., 2013; Therit et al., 2015). The VirS/VirR bicomponent signal transduction system indirectly upregulates the expression of *nanI* and *nanJ* (Ohtani et al., 2010), where VirS first upregulates the expression of the *vrr* gene (encoding an RNA (VR-RNA) regulated by VirR), and then this regulatory RNA regulates the expression of sialidase-encoding genes (Ohtani et al., 2010). The RevS/RevR system directly or indirectly positively regulates *nanJ* expression and negatively regulates that of *nanI*. However, since more than 100 genes are differentially expressed in *revR* mutants, the regulation of sialidase genes by this system is likely indirect (Hiscox et al., 2011).

Abe et al. (2010) observed that an RNA binding protein called Tex (CPE2168) affected *nanJ* mRNA levels in a non-sialic acid dependent manner. However, it is important to note that the results obtained with *tex* mutant have not been confirmed by complementation. And another study reported that the expression of *nanI* gene was up-regulated in both a CPE1446 mutant and a CPE1447-CPE1446 deletion mutant. Complementation of CPE1446 and CPE1447 revealed that a heterologous complex of the proteins CPE1446 and CPE1447 negatively regulated *nanI* expression in a non-sialic acid-dependent manner (Obana and Nakamura, 2011). In the absence of sialic acid in the growth medium, the ReeS/ReeR system positively regulates the transcription of *nanI* and *nanJ* (Hiscox et al., 2013). Therefore, there are additional sialic acid specific transcription regulatory factors that have yet to be identified in *C. perfringens*.

NanR is a member of the RpiR transcription factor family. NanR finely regulates the generation and use of sialic acids to support sporulation and CPE production (Mi et al., 2018). There are six NanR binding sites in the *nanI* promoter region (Therit et al., 2015). When *C. perfringens* is grown in the absence of sialic acid, NanR binds to some or all of the binding sites in the *nanI* promoter. NanR represses the production of NanI and the expression of sialic acid metabolism-related proteins (Therit et al., 2015). However, because there are similar NanR binding sites near the *nan* operon, NanR may reduce the expression of this operon in the absence of sialic acid (Mi et al., 2018). In the presence of free sialic acid, sialic acid from glycoconjugates is metabolized by *C. perfringens* to produce ManNAc-6P, which binds to NanR to relieve the inhibition of *nanI* expression (or possibly that of the *nan* operon). Another unknown repressor that may reduce *nanI* expression in the presence of high concentration of sialic acid. Moreover, the expression of *nanI* decreases in the presence of high glucose concentrations, suggesting that in addition to NanR, there is an additional repressor that represses *nanI* expression. Whether this repressor is the same as that repressing the expression of *nanI* in the presence of a high concentration of sialic acid remains to be determined (Li et al., 2017). Since NanI can affect the expression of *ccpA* and *codY* (Li et al., 2015), the CcpA and CodY regulatory proteins may be candidates for the aforementioned *nanI* repressors, but that remains to be verified. Furthermore, an investigation using a *codY* null mutant of the type D *C. perfringens* strain CN3718 showed that CodY repressed the production of NanJ and NanH, while failing to affect the production of NanI (Li et al., 2013). Thus, sialidases are regulated by multiple systems, and the relationship between these systems awaits further elucidation.

## Potential Role of Sialidases in *C. Perfringens* Pathogenesis

### Effects of Sialidase on *C. perfringens* Growth and Survival

The ability of bacteria to grow in the gut requires tenacity, as the competition for nutrition among intestinal microbes is extremely intense (Tailford et al., 2015). Particularly during intestinal diseases, the damaging effect of diarrhea can also reduce the concentration of enteral nutrients. One of the coping strategies bacteria use to respond to limited enteral nutrients is the production of sialidases (Severi et al., 2007), which enable sialic acid to be obtained from the host as a source of carbon, nitrogen, amino acids, and energy. Sialic acid is typically transported into bacterial cells by a sialic acid transporter (NanT), sialic acid-specific subfamily of TRAP transporters (SiaPQM), or an ATP binding transporter (SatABCD), after which it is metabolized to fructose-6-P (Vimr et al., 2004).

*C. perfringens* was one of the first bacteria to have been shown to be able to use sialic acid as a carbon source (Nees and Schauer, 1974). NanI and NanJ have been shown to cause Caco-2 cells (human intestinal-like cells) to release sialic acid, while NanH does not have this activity (Li and McClane, 2014b). A recent study (Li and McClane, 2018) has shown that *C. perfringens* strain F4969, a non-food-borne human intestinal infectious strain, can use mucin or Caco-2 cells to support its growth and survival. In addition, natural levels of NanI can enhance the growth and survival of bacteria in the gut, in part due to the ability of NanI to release free sialic acid from mucin or sialic acid-modified macromolecules from host cells to support bacterial survival. Furthermore, NanI exposes the underlying carbohydrates and amino acids by removing the terminal sialic acids from macromolecules, allowing other glycoside hydrolases or proteases to hydrolyze and release nutrients for bacterial growth and utilization. However, NanI is not a major contributor to the early logarithmic growth of this strain. When bacteria enter the late logarithmic growth period and encounter more restricted nutrient levels, they seek alternative nutrients by increasing the production of sialidases, especially NanI. Moreover, free sialic acids produced by NanI allow other enzymes to produce carbohydrates and amino acids for bacterial growth. Thus, other factors may act synergistically with NanI to enhance the growth of *C. perfringens* (Li and McClane, 2018).

Previous *in vitro* studies have shown that NanI promotes the growth and survival of bacteria, facilitating bacterial colonization in the gut (Li and McClane, 2014b). A recent *in vivo* study showed that the type F NFD strain F4969 could survive for at least 4 days in the small intestine, caecum, and colon of mice (Navarro et al., 2018). However, when the mice were infected with the *nanI* mutant, the number of *nanI* mutant recovered from each intestinal segment was significantly lower than that observed for the wild-type strain. In addition, the mutants were completely cleared from the small intestine on the 4th day, while the complementation of the *nanI* mutation restored the colonization ability of this strain. When mice were inoculated simultaneously with the same *nanI* null mutant and a *nanI*-producing strain, the number of mutant bacteria in any of the assayed intestinal segments returned to the level of the wild-type strain F4969. These results showed for the first time that NanI is an important contributor for the colonization of NFD strains in the intestinal tract, clearly demonstrating that sialidases produced by bacterial pathogens can enhance the colonization of bacteria in the intestinal tract and are an important contributor to chronic intestinal infections (Navarro et al., 2018). This result also provides a reasonable explanation for the presence of the NanI gene in *C. perfringens* strains that persist and colonize the intestinal tract as well as the absence of the *nanI* gene in *C. perfringens* type F strains carrying a chromosomal *cpe* gene, which cause acute infections.

Although some roles for sialidases in intestinal infections caused by *C. perfringens* have been elucidated, the effect of sialidase on the growth and colonization of *C. perfringens* in parenteral infections is unclear. A study showed that a *nanJ* and *nanI* double null mutant of *C. perfringens* strain 13 remained virulent in a murine myonecrosis model (Chiarezza et al., 2009). This result indicates that Neu5Ac metabolism is not necessary for the growth or colonization of *C. perfringens* in muscle tissue (Chiarezza et al., 2009). However, this result could not exclude the possibility of some subtle effects of sialidases on the colonization or growth of *C. perfringens* because the mouse myonecrosis model requires a large number of inoculum, which may mask subtle effects (Chiarezza et al., 2009). Thus, the role of sialidases in parenteral infections caused by *C. perfringens* awaits further study.

### Effects of Sialidase on *C. perfringens* Adhesion

The type D *C. perfringens* strain CN3718 adheres weakly to fibroblasts and renal cells, whereas this strain can adhere to cultured Caco-2 cells. Compared with a triple mutant strain that does not produce any sialidases, the wild-type strain CN3718 exhibits significantly increased adhesion to Caco-2 cells. Complementation studies showed that, of the three sialidases made by CN3718, restoring NanI production to the triple sialidase mutant yielded the greatest enhancement of adherence. Thus, NanI can significantly promote the specific adhesion of the CN3718 strain to Caco-2 cells and plays a major role in enhancing the adhesion of this strain to Caco-2 cells among the three sialidases (Li et al., 2011).

The process of *C. perfringens* adhesion to host cells can be divided into two steps. First, secreted NanI specifically modifies the surface of host cells, after which an unknown adhesin on the surface of *C. perfringens* binds to an unknown receptor on the surface of intestinal epithelial cells. The affinity of the *C. perfringens* CN3718 adhesin to some mammalian cells (such as Caco-2 and HT-29 intestinal cells) is higher than that observed to non-intestinal cell lines (Li et al., 2011). Thus, NanI can modify the surface of host cells to specifically increase the adhesion of CN3718.

Sialidases also non-specifically enhance the adhesion of *C. perfringens* to host cells, primarily due to the negative charges of sialic acids at the distal end of carbohydrate chains (Severi et al., 2007; Lewis and Lewis, 2012). Moreover, terminal sialic acids can destroy the integrity of the endothelial barrier. The treatment of monolayers of epithelial cells with *C. perfringens* sialidases results in barrier damage, which promotes the ability of *C. perfringens* to approach and adhere epithelial cells (Cioffi et al., 2012). The non-specific interaction between secreted NanI and electrical charges associated with the an intact epithelial barrier contributes to the enhancement of toxin binding and the colonization of *C. perfringens*.

Studies on the mechanisms of the adherence of *C. perfringens* to host cells and tissues are very limited (Jost et al., 2006; Hitsumoto et al., 2014; Katayama et al., 2015). Although NanI can significantly promote the specific adhesion of CN3718 to Caco-2 cells, NanI itself is not the major adhesin (Li et al., 2013). Thus, the adhesin used by *C. perfringens in vivo* remains unclear.

### Effects of Sialidases on the Cytotoxicity of *C. perfringens* Toxins

In addition to affecting bacterial colonization, NanI can enhance the activity of α-toxin that causes gas gangrene (Flores-Díaz et al., 2005; Chiarezza et al., 2009), while also increasing the cytotoxicity of β- and ε-toxins and NetF and CPE toxin associated with gastrointestinal diseases (Li et al., 2011; Mehdizadeh Gohari et al., 2018; Theoret et al., 2018). Although many *C. perfringens* pathogenic strains only produce a small amount of CPB, CPE, or ETX (Collie et al., 1998; Sayeed et al., 2005; Fisher et al., 2006; Fernandez-Miyakawa et al., 2007; Ma et al., 2014), and the enhancement effect of natural levels of NanI on CPB, CPE and ETX is relatively moderate (1.5–2-fold), this activity may be important for the pathogenesis of *C. perfringens* during enteritis or enterotoxemia. The primary mechanism by which NanI enhances the cytotoxicity of CPB, CPE, and ETX is to enhance the binding of toxins to host cells. Because CPE, CPB, and ETX do not share the same receptors, the enhancement of the binding of toxins to host cells induced by NanI is essentially non-specific. This enhancement may involve one or more mechanisms, one of which is that most of the charges on mammalian cell surfaces are provided by sialic acids, and NanI reduces electrostatic repulsion and enhances toxin binding by removing surface sialic acid residues. Alternatively, NanI may facilitate the accessibility of the toxins to their receptors by removing sialic acids from the toxin receptors. A third possibility is that NanI may enhance toxin binding by trimming glycoproteins or glycolipids adjacent to toxin receptors, removing some of the sialic acids that block the toxin receptors. In addition, sialidases can increase paracellular permeability (Cioffi et al., 2012). Finally, NanI makes toxins more accessible to receptors on the basolateral surface of host cells, leading to increased toxin binding. The mechanism(s) by which NanI enhances the cytotoxicity of CPB, CPE, and ETX requires additional investigations (Theoret et al., 2018).

### Hydrolysis and Activation of Sialidases

When intestinal diseases occur, the proteins such as ETX and CPE toxins secreted by *C. perfringens* contact host proteases (such as trypsin) in the intestinal cavity and become hydrolyzed and activated (Hanna et al., 1992; Freedman et al., 2014). NanI can also be hydrolyzed and activated by trypsin, whereas NanJ can't be activated. The activation of NanI by trypsin may promote its activity (Li et al., 2011; Li and McClane, 2014a). The activation of trypsin by NanI promotes intestinal diseases caused by *C. perfringens* (Theoret et al., 2018). Recently, purified chymotrypsin has been shown to activate NanI, and it is worth mentioning that small intestine fluid can activate NanI *in vitro*. During the course of intestinal diseases, NanI is produced in the gastrointestinal tract and is activated through contact with fluid in the small intestine that contains a mixture of intestinal proteases (including trypsin and chymotrypsin). The hydrolysis and activation of NanI can further enhance the effects of CPE, ETX, and CPB, promoting the occurrence of disease (Theoret et al., 2018). Thus, the hydrolysis and activation of NanI may contribute to the pathophysiology of *C. perfringens* (Theoret et al., 2018). Therefore, NanI is a potential auxiliary virulence factor promoting enteritis and enterotoxemia caused by *C. perfringens*.

## Sialidase Inhibitors

Sialidases have become a drug target for the treatment of viral and bacterial infections (Soong et al., 2006; Memoli et al., 2008). Some studies have shown that a number of sialidase inhibitors may become effective drugs for the treatment of sialic acid toxicosis, cancers, infections, immune diseases, atherosclerosis, and many other diseases (Karagodin et al., 2018). Recently, researchers have reported encouraging findings. One study showed that sialidase inhibitors could attenuate pulmonary fibrosis in mice, suggesting that sialidase inhibitors may be helpful in the treatment of fibrosis (Karhadkar et al., 2017). Another study indicated that C9-BA-DANA inhibits endogenous and ectopically expressed sialidase activity and established NEU1-mediated bioactivities in human airway epithelia, lung microvascular endothelia, and fibroblasts *in vitro* and murine lungs *in vivo* (Hyun et al., 2016).

Two classical inhibitors of *C. perfringens* sialidases, Siastatin B (SB), and N-acetyl-2,3-dehydro-2-deoxyneuraminic acid (NADNA), have been shown to inhibit *C. perfringens* sialidase activity (Li and McClane, 2014a,b; Li et al., 2015). SB or NADNA can reduce the adhesion of *C. perfringens* AAD strain F4969 to Caco-2 cells (Li and McClane, 2014b) and effectively inhibit the activity of sialidases in cell-free supernatants collected from *C. perfringens* D strain CN3718 cultures. Moreover, SB can reduce the production of ETX in strain CN3718 (Li et al., 2015). A recent study showed that SB reduced the growth and survival rate of strain F4969 in the presence of Caco-2 cells (Li and McClane, 2018). Diseases caused by *C. perfringens* are challenging to treat with antibiotics since toxins that have already been synthesized continue to work after the administration of antibiotics. Similarly, because some strains produce many different toxins, it is often difficult to treat or prevent *C. perfringens* infections with vaccines or neutralizing antibodies (Li et al., 2015). Although NanI can enhance *C. perfringens* adherence and toxin binding, it is worth mentioning that NanI would not be needed for nutritional purposes or to enhance bacteria adherence or colonization during the acute food poisoning (Li and McClane, 2014b). So sialidase inhibitors may be a potential candidate for the development of drugs against human non-foodborne gastrointestinal disease such as antibiotic-associated diarrhea and sporadic diarrhea, and the development of highly effective sialidase inhibitors will be an important direction in generating anti-*C. perfringens* infection drugs in the near future.

The development of targeted sialidase inhibitors is based on random screening of synthetic compounds or substrate (sialic acid) mimics. In recent years, researchers have studied natural non-substrate mimic sialidase inhibitors, such as flavonoids. Natural flavonoids have potential in promoting resistance to trypanosomiasis (Arioka et al., 2010). Research has shown that these compounds also have a significant competitive inhibitory effect on *C. perfringens* sialidases (Cp-NanI) (Lee et al., 2014).

**Figure 4:**
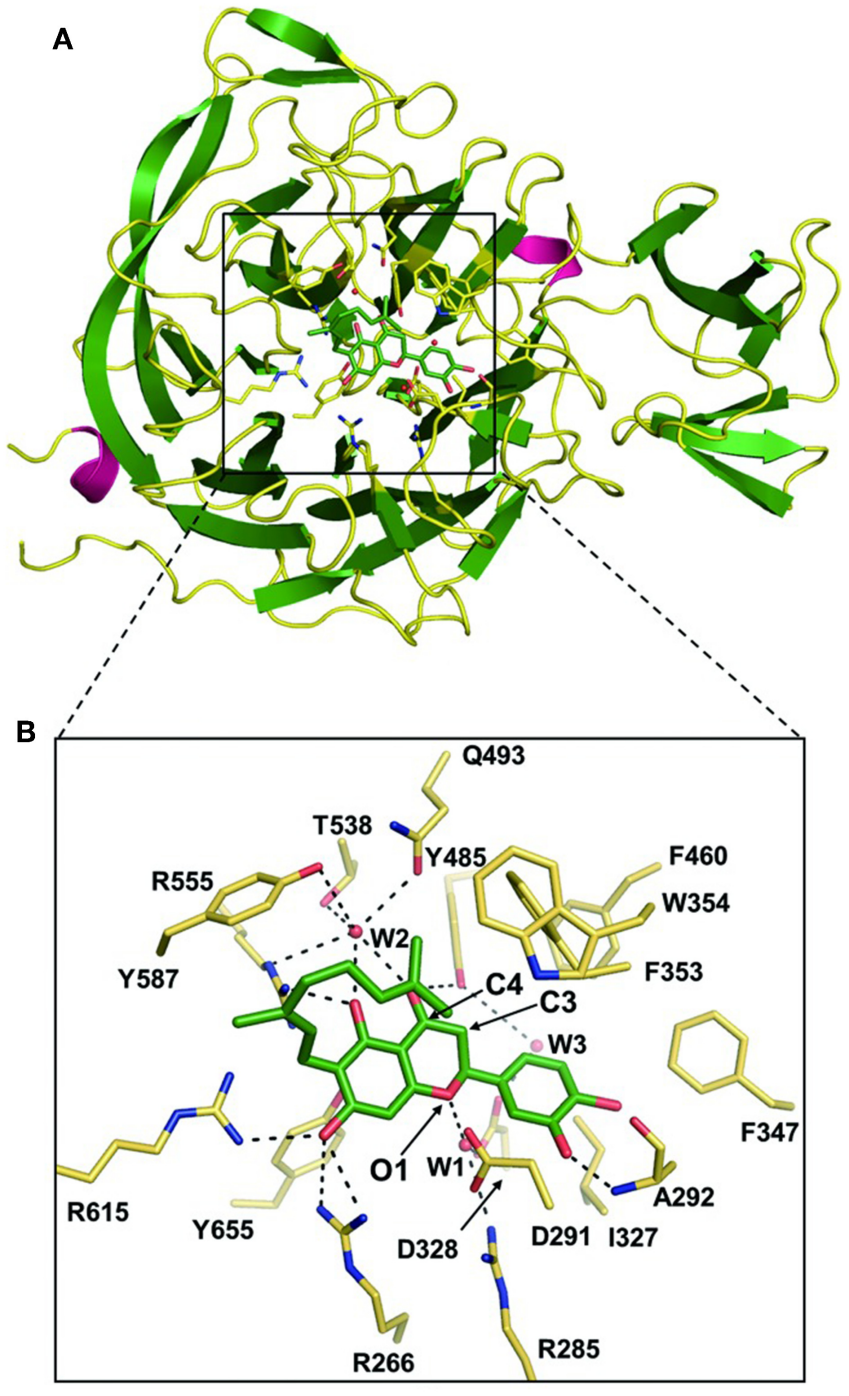
The Cp-NanI catalytic site with diplacone. Reprinted from Lee et al. (2014), Copyright © (Lee et al., 2014). **(A)** Overall structure of the Cp-NanI catalytic domain bound to diplacone. **(B)** Details of the mode of binding. Diplacone and three water molecules (W1, W2, and W3) are shown as green sticks and red spheres, respectively. Hydrogen bonds are displayed as dashed lines.

Biochemical and structural data on sialidases can provide valuable information for the design of new selective antimicrobial or antiviral drugs. A recent study has identified the crystal structure of the Cp-NanI catalytic domain (residues 243-694) complexed with diplacone and elucidated their interaction in detail. The interactions between the inhibitor and the enzyme are primarily hydrogen bonds and hydrophobic interactions. The 3′,4′,5′,7′-hydroxy-4-oxo group and O atom at the 1-position of diplacone forms direct and indirect hydrogen bonds with the tri-Arg cluster (Arg266, Arg555, and Arg615), Arg285, Asp328, Phe353, Tyr485, Gln493, Thr538, and Tyr655 residues on the side chain and the Ala292 residue on the primary chain, as well as with three water molecules (Figure 4). The flavanone backbone of diplacone and the Ile327, Phe347, Phe460, Tyr485, and Tyr655 residues correctly position the conformation of the stable inhibitor complex through hydrophobic interactions (Lee et al., 2014).

Microorganism drug resistance remains a difficult problem. But what's exciting is that studies found various natural flavonoids contain flavanone backbones, which have an inhibitory effect on viral sialidases (Grienke et al., 2012). This finding may aid in solving the problem of sialidase inhibitor resistance, because regardless of the I223R or H275Y mutation, diplacone appears to have anti-sialidase activity. As with the wild-type protein, the C4 hydroxyl group of diplacone can interact with the Glu276 of the H275Y mutant, suggesting that diplacone may become a potential lead compound for the development of inhibitors for viral sialidase drug resistant mutants (Lee et al., 2014). Therefore, the elucidation of the structural information of the Cp-NanI-diplacone complex is extremely helpful in the development of antibacterial and antiviral sialidase inhibitors.

At present, the development of new natural antibacterial sialidase inhibitors has become a novel direction of research. Kim et al. (2018) focused on the natural product turmeric and observed that a curcumin derivative compound, 7-(3,4-dihydroxyphenyl)-5-hydroxy-1-(3-hydroxy-4-methoxyphenyl) hepta-1,4,6-trien-3-one, inhibits *S. pneumoniae* NanA and sialidases from *V. cholerae* and *C. perfringens*. The methoxy or hydroxyl group, heptadienyl, and α, β unsaturated ketone groups play important roles in the activity of this inhibitor. Although the positions of the methoxy and hydroxyl groups do not affect the inhibitory effects on the enzymes, the inhibition increases with the increase in the number of hydroxyl groups substituted in benzene rings. The activity of the *C. perfringens* sialidase inhibitor is not affected by an electronic effect, and the catechol moiety group in this compound plays an important role in enhancing its inhibitory effect.

## Conclusion and Prospection

Sialidases, especially NanI, are emerging as potential pathogenic factors during *C. perfringens* intestinal infections. These enzymes can function by upregulating the production of toxins associated with intestinal infections, enhancing the cytotoxicity of toxins, increasing the adhesion of *C. perfringens* to host cells, and producing substrates used for bacterial growth and metabolism (Li and McClane, 2014a; Li et al., 2015). In the past, studies of *C. perfringens* virulence were focused on toxins. Although studies on the functions of sialidases in the pathogenesis of *C. perfringens* have yielded interesting results in recent years, there are still many questions to be answered. Among the questions to be answered is whether NanI directly contributes to disease. Future studies on *C. perfringens* sialidases may also involve investigating the following aspects: (1) the relationship between various systems involved in the regulation of sialidases; (2) the roles of sialidase NanH and NanJ in intestinal infections caused by *C. perfringens* and whether NanH, NanI, and NanJ have synergistic effects; (3) the differences in the specific sialidases produced by epidemiologically different strains of *C. perfringens*. And whereas the catalytic mechanism of the *S. pneumoniae* sialidase NanC has been reported (Xiong et al., 2018), those of the *C. perfringens* sialidases have not been reported. To facilitate the design and development of sialidase inhibitors in the future, studies on the catalytic mechanism of sialidases are required. In addition, the inhibition of sialidase activity in *P. gingivalis* renders the bacteria easier to be cleared by macrophages (Yang et al., 2018). Studies on this aspect of the *C. perfringens* sialidase have not been reported and should therefore be a major focus of future studies. Thus, the continued study of sialidases will promote new breakthroughs in the prevention and treatment of patients suffering infections by *C. perfringens* in the near future.

## Author Contributions

Y-HW collected the references and wrote the paper.

### Conflict of Interest

The author declares that the research was conducted in the absence of any commercial or financial relationships that could be construed as a potential conflict of interest.
